# Lettuce be happy: A longitudinal UK study on the relationship between fruit and vegetable consumption and well-being

**DOI:** 10.1016/j.socscimed.2018.12.017

**Published:** 2019-02

**Authors:** Neel Ocean, Peter Howley, Jonathan Ensor

**Affiliations:** a University of Leeds, UK; b Stockholm Environment Institute, University of York, UK

**Keywords:** UK, Well-being, GHQ-12, Fruit and vegetables, Diet, UKHLS, Panel data, Fixed-effects

## Abstract

**Rationale:**

While the role of diet in influencing physical health is now well-established, some recent research suggests that increased consumption of fruits and vegetables could play a role in enhancing mental well-being. A limitation with much of this existing research is its reliance on cross-sectional correlations, convenience samples, and/or lack of adequate controls.

**Objective:**

We aim to add to the emerging literature on the relationship between fruit and vegetable consumption and well-being by using longitudinal data from a study in the United Kingdom (UK).

**Method:**

We employ panel data analytical techniques on three waves collected between 2010 and 2017 (i.e., following the same individuals over time) in the UK Household Longitudinal Survey. We also control for time-variant confounders such as diet, health, and lifestyle behaviours.

**Results:**

Fixed effects regressions show that mental well-being (GHQ-12) responds in a dose-response fashion to increases in both the quantity and the frequency of fruit and vegetables consumed. This relationship is robust to the use of subjective well-being (life satisfaction) instead of mental well-being. We also document a hump-shaped relationship between fruit and vegetable consumption and age.

**Conclusion:**

Our findings provide further evidence that persuading people to consume more fruits and vegetables may not only benefit their physical health in the long-run, but also their mental well-being in the short-run.

## Highlights

• Increased fruit & vegetable consumption can enhance mental well-being.
• Increasing frequency and increasing quantity of consumption both matter.
• The relationship is robust to different measures of well-being.
• A hump-shaped relationship appeared between age and fruit and vegetable consumption.

## 1 Introduction

A recent development in the well-being literature has been to show that increased consumption of fruits and vegetables are positively associated with mental and subjective well-being. One of the first studies in this area used large representative samples from the Welsh Health Survey, the Scottish Health Survey, and the Health Survey of England to demonstrate a positive association between fruit and vegetable consumption and psychological well-being (Blanchflower et al., 2013). More recently, Lalji et al. (2018) also used the Health Survey of England to examine the association between fruit and vegetable consumption and health. In comparison to Blanchflower et al. (2013) they examined a much broader range of health outcomes such as blood pressure and levels of cholesterol, in addition to psychological well-being. They found that individuals who consumed three to four portions of fruit daily reported better psychological well-being compared to those who did not.

Further recent cross-sectional studies suggestive of a link between fruit and vegetable consumption and well-being include Peltzer and Pengpid (2017) and Sapranaviciute-Zabazlaieva et al. (2017). Respectively, these studies found a positive link between fruit and vegetable consumption and mental well-being amongst: university students across 28 countries; and for 45-72 year-olds living in Lithuania. A further cross-sectional study found that the relationship is stronger for raw fruit and vegetables than those consuming them cooked or canned (Brookie et al., 2018). While all of these findings were robust to adjustment for a large number of economic, social, and demographic variables, the main limitation (as pointed out by the authors) is that confounding remains possible when the data are cross-sectional.

An innovative psychology paper from the same year as the original cross-sectional study by Blanchflower at al. goes some way to tackling the potential bias from unobserved confounders. In this study a ‘daily diary’ survey was undertaken with young adults (White et al., 2013): Uniquely, the same individuals (*n* = 281) were followed over a period of 21 consecutive days. Well-being was measured in terms of positive and negative affect, rather than general health or life satisfaction. On a day where positive affect was one point higher (on a 1 to 5 scale) for an individual, that individual reported consuming 0.112 more portions of fruit (*p* = 0.002) and 0.147 more portions of vegetables (*p* < 0.001). In addition, using lagged variables revealed that fruit and vegetable consumption predicts positive affect the following day, but positive affect *did not* predict fruit and vegetable consumption. This is important as it suggests fruit and vegetable consumption leads to higher well-being, but higher well-being does not lead to higher fruit and vegetable consumption.

A subsequent study with a similar design (Conner et al., 2015) found that fruit and vegetable consumption also positively predicts eudaimonic well-being (i.e., engagement, purpose, and meaning), creativity, and curiosity. Whereas the studies by White et al. (2013) and Conner et al. (2015) were able to track the same individuals over time (which is advantageous when it comes to establishing causality), they utilised a convenience sample consisting of psychology students, which is small in number relative to Blanchflower et al. (2013). The cross-sectional and diary approaches taken together do, however, strongly support the suggestion that diet could be an important factor when it comes to explaining variation in well-being.

A significant step towards demonstrating the causality of this relationship came from a randomised control trial by Conner et al. (2017), and a panel data analysis by Mujcic and Oswald (2016). Conner et al. (2017) found that various psychological outcomes improved significantly in a treatment group which provided young adults two additional portions of fruit and vegetables a day over a two-week period. Though this study provided an important step towards establishing causality, it is limited by a relatively small sample of 171 18-25 year-olds, and a short time frame (two weeks).

Mujcic and Oswald (2016) used individual level panel data - namely the nationally representative and longitudinal Household, Income, and Labour Dynamics in Australia (HILDA) survey – to examine the relationship between fruit and vegetable consumption and subjective well-being. The unique feature of this dataset, relative to the cross-sectional work described above, is that the same individuals could be tracked between two points in time. Using individual level fixed-effects, Mujcic and Oswald (2016) found that the average number of fruit and vegetable portions consumed in one day positively and significantly predicts life satisfaction, as well as an alternative happiness measure. What is particularly surprising is that this relationship appears to be fairly linear and monotonically increasing. In other words, the benefits from increased daily fruit and vegetable consumption do not appear to diminish, even when consumption is in excess of the five portions per day often recommended in a number of developed countries (including the UK, US, and Japan).

This study aims to build on this research which is suggestive of a positive relationship between fruit and vegetable consumption and well-being. We use a similar panel-data estimation approach to Mujcic and Oswald (2016), with two main differences. First, we use data from a much larger representative longitudinal survey in the UK, namely the UK Household Longitudinal Survey (UKHLS). Similarly to Mujcic and Oswald (2016), the structure of this survey allows us to examine the relationship between daily variation in the consumption of fruit and vegetables and well-being, but we are also able to ascertain estimated effects for fruit and vegetable consumption separately when it comes to frequency of consumption, i.e., how many days in a week an individual typically consumes fruits and vegetables. Second, as well as using a standard subjective life satisfaction measure (similar to the one used by Mujcic and Oswald, 2016), we also use a measure of ‘mental well-being’: the 12-item General Health Questionnaire (GHQ-12). This measure, aside from being finer-grained, is also more relevant in capturing changes in mental health and thus perhaps better suited for informing public health policymakers. Finally, we also examine to what extent fruit and vegetable consumption in the UK varies along socio-demographic lines.

The key advantage associated with the longitudinal nature of our dataset (and the one used by Mujcic and Oswald) is that we can relate changes in fruit and vegetable consumption to changes in self-reported well-being for the same individual over time (fixed-effects). Doing so helps ensure that any observed relationship is not the result of a spurious cross-sectional pattern caused by the potentially pernicious effect of time-invariant omitted confounders such as personality traits or family upbringing. In addition to controlling for the standard set of socio-demographic variables, we also control for a variety of time-variant confounders reflective of health, diet and lifestyle behaviours that could plausibly bias any estimates relating to the relationship between fruit and vegetable consumption and well-being.

Using two separate indicators, our results strongly suggest that fruit and vegetable consumption can enhance well-being. Specifically, using the General Health Questionnaire and self-reported life satisfaction as our indicators, we find that well-being rises in an approximately dose-response way with both the number of portions of fruits and vegetables consumed, and the number of days in a given week an individual consumes either fruits or vegetables. Our findings, therefore, provide further evidence that persuading or incentivising people to consume more fruits and vegetables may not only benefit their physical health in the long run, but also their mental well-being in the short run.

## 2 Methods

In order to examine the relationship between well-being and the consumption of fruit and vegetables, we draw upon the UK Household Longitudinal Study (UKHLS) – also known as ‘Understanding Society’. The UKHLS contains information from approximately 50,000 individuals and is the replacement for the older British Household Panel Survey (BHPS). Data for ‘mainstage waves’ 1–7 were collected from 2009 to 2017.

Households recruited in the first wave from a general population sample across the whole of the UK were visited annually, and responded either via online survey or a face-to-face home interview conducted by a trained interviewer. This mixed mode of response is designed to increase response rates. Household response rates for the three waves (2, 5, and 7) used in this study are 76.2%, 83.8%, and 81.5% (understandingsociety.ac.uk/sites/default/files/downloads/documentation/mainstage/user-guides/mainstage-waves-1-7-user-guide.pdf, last accessed on 11th Sep 2018). Respondents of the main survey are household members aged 15 or over (individuals between 10 and 15 complete a separate youth questionnaire). Not all questions (such as those relating to fruit and vegetable consumption) are asked each year, in order to lessen the burden on participants.

### 2.1 Variables

We identified three questions on fruit and vegetable consumption that allow us to estimate the impact of both quantity and frequency. In order to measure quantity, we analysed responses to the following question: “On a day when you eat fruit or vegetables, how many portions of fruit and vegetables in total do you usually eat?” The size of a portion is defined as being equal to: one cup of raw vegetables (or the size of a fist), half a cup of cooked vegetables or chopped fruit, or one piece of fruit. Individuals were also shown pictures to help define portion size. In order to measure frequency, two separate questions asked respondents how often they consumed fruit and how often they consumed vegetables in a usual week. Both of these items were measured categorically, with four categories: *Never*; *1–3 days per week*; *4–6 days per week*; *Every day*.

Data on the number of fruit and vegetable portions consumed on a typical day where there is non-zero consumption of fruit and vegetables is available in Waves 2 and 5 of the UKHLS, while observations on the frequency of fruit and vegetable consumption in a typical week were available in Waves 2, 5, and 7. Data for Wave 2 were collected between Jan 2010 and June 2012; Wave 5 was collected between Jan 2013 and June 2015; and Wave 7 was collected between Jan 2015 and June 2017. Unlike Mujcic and Oswald (2016), we chose not to combine the frequency and quantity measures into one estimate of average daily fruit and vegetable consumption, and instead kept them separate. Doing so allowed us to separate frequency and quantity effects, and also to exploit the additional wave of data for frequency effects. The data, therefore, enable us to measure quantity effects between two time points spanning three years, and frequency effects across three time points spanning five years.

Our main outcome variable is mental well-being, measured using the GHQ-12. The General Health Questionnaire (GHQ) measure was conceived as a screening instrument for psychiatric disorders (e.g., see Goldberg and Hillier, 1979), but is also used as a general measure of mental well-being. The GHQ-12 is a shortened version of the original GHQ, which consists of 60 items instead of 12. The original 60-item GHQ measure is still used when more intensive examination is required on smaller numbers of individuals. The items of the GHQ-12 encompass aspects of hedonic well-being (feeling happy or depressed), eudaimonic well-being (purpose and self-worth), and anxiety (worry and strain). Each item is scored on a four-point Likert scale, whose options are coded from 0 to 3. Therefore, the GHQ-12 score ranges from 0 (best well-being) to 36 (worst well-being). For ease of interpretation, we reversed this variable so that individuals are scored from 0 (worst) to 36 (best). In the regression tables that follow, we labelled this variable as ‘*reversed GHQ-12*’. Therefore, a higher level of *reversed GHQ-12* corresponds to a higher level of mental well-being. As a useful robustness check, we also used an alternative measure of subjective well-being as an outcome variable, namely self-reported life satisfaction.

Based on prior research, we included a rich set of commonly observed predictors of well-being as control variables (see Dolan et al., 2008 for a review of this literature). These include socio-demographic variables such as age, household income, gender, relationship status, number of children and education. Such controls are important as it is possible that fruit and vegetable consumption would co-vary with, for example, income – someone becoming richer and as a result enjoying higher well-being scores and changing their food related behaviours. We also controlled for the presence of long-standing health conditions as well as health and lifestyle related behaviours such as walking frequency and smoking behaviour – again potential confounders that could bias any estimates of the relationship between fruit and vegetable consumption and well-being. Long-standing health conditions were measured by responses to the following question: *“Do you have any long-standing physical or mental impairment, illness, or disability? By 'long-standing' I mean anything that has troubled you over a period of at least* 12 months *or that is likely to trouble you over a period of at least* 12 months*.”* Walking frequency was measured in terms of the number of days on which an individual walked continuously for 10 min or more in the last 4 weeks.

Finally, we added other controls reflective of food related behaviours, namely the type of bread and milk consumed by individuals. The type of bread eaten most frequently could be either: white; wholemeal; granary or wholegrain; other brown; both brown and white; other; or none. The type of milk usually consumed could be either: whole; semi-skimmed; skimmed; soya; other; or none. These serve as useful proxy variables for overall dietary patterns. That is, someone consuming semi-skimmed milk or wholegrain bread may be more conscious of diet than someone consuming full fat milk or regular white bread.

### 2.2 Estimation strategy

Our main regression analysis seeks to estimate the relationship between the quantity of fruit and vegetable portions consumed (on a day where at least one portion is consumed) and mental well-being, as captured by our *reversed GHQ-12* measure. Specifically, we estimated this equation:

$$W_{it}=\beta_{0}+\beta_{1}X_{it}+\beta_{2}E_{it}+\;\beta_{3}H_{it}+\;\;\beta_{4\;}FV_{it}\;+\;a_{i}+v_{t}+\;\varepsilon_{it}$$

where $W_{it}$ is our measure of individual mental well-being (*reversed GHQ-12*), $v_{t}$ is the time (wave) dummy, and $\varepsilon_{it}$ is an idiosyncratic error term. $FV_{it}$ is our key explanatory variable, which reflects daily levels of fruit and vegetable consumption. The vector $X_{it}\;$ is a standard set of socio-demographic control variables that might be correlated with individual well-being (e.g., income, age, education, labour force status, relationship status etc.). $E_{it}$ captures other lifestyle behaviours that could be related with both well-being and fruit and vegetable consumption (e.g., exercise measured by walking frequency, other dietary habits, and smoking behaviour). $H_{it}$ captures whether individuals have a long-standing health condition. Finally, $a_{i}$ represents the individual fixed-effect, which controls for any time-invariant unobserved confounders (e.g., personality traits and differences in family upbringing).

In addition to examining the relationship between the *number* of fruit and vegetable portions consumed on a day that an individual consumes some fruit and vegetables, and mental well-being, we estimated a reduced-form model where we explore the relationship between the *frequency* (i.e., number of days) with which fruits and vegetables separately are consumed in a typical week and mental well-being. For this analysis, we were able to exploit three waves of data. It is a reduced form model, as some of our controls (e.g., exercise and smoking behaviour) were not available across all three waves. They were therefore excluded from this part of the analysis.

The outcome variable used in these analyses is mental well-being, measured by *reversed GHQ-12*. As an additional robustness check, we repeated these analyses using a self-reported measure of life satisfaction as the dependent variable. This was reported on a 7-point Likert scale ranging from ‘completely dissatisfied’ to ‘completely satisfied’. We used this alternative measure because such self-reported life satisfaction measures are commonly used in the literature, and so we thought it would be a useful exercise to check whether the overall qualitative interpretation of our main results is sensitive to the well-being measure used.

## 3 Results

Our results are split into three main sections. We first provide some descriptive statistics relating to the extent to which fruit and vegetable consumption varies according to socio-demographic characteristics. Second, we present the results of our main fixed-effects analysis, which examines the relationship between fruit and vegetable consumption and mental well-being as captured by *reversed GHQ-12*. Third, as a robustness check, we examine whether our main results are sensitive to an alternative metric of well-being (namely self-reported life satisfaction).

### 3.1 Descriptive statistics

**Fig. 1:**
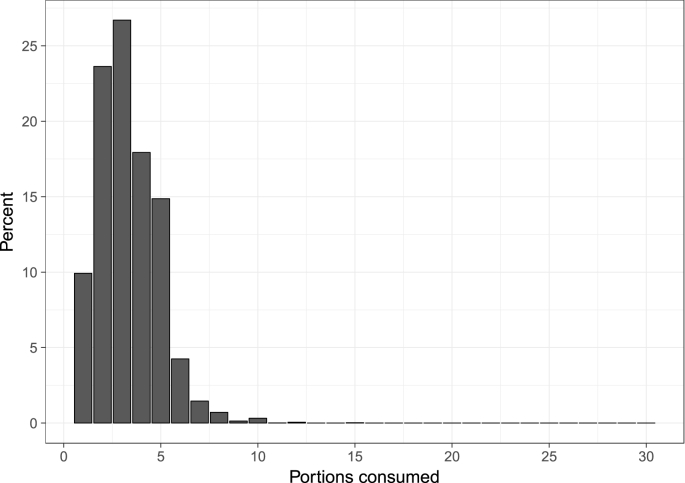
Number of fruit and vegetable portions consumed on a day when at least one portion is consumed (*n* = 90,448).

**Fig. 2:**
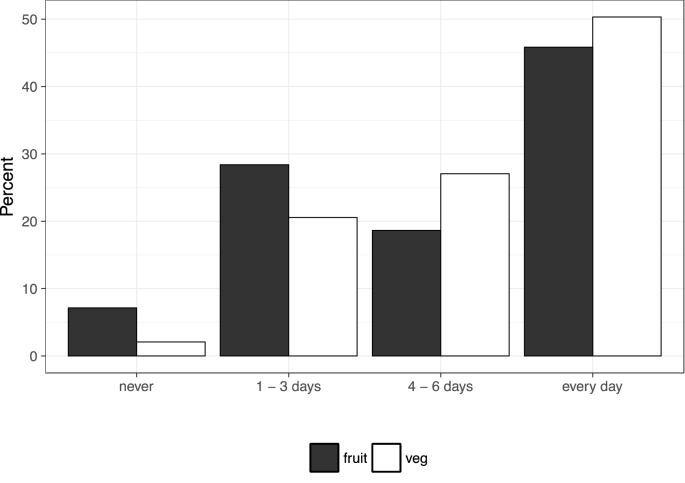
The number of days per week where a non-zero amount of fruit (*n* = 130,547) or vegetables (*n* = 130,557) is consumed.

Fig. 1 shows the number of fruit and vegetable portions consumed in a day when there is a non-zero level of consumption (our quantity measure), that is, on a day when fruits and/or vegetables are consumed. An interesting observation is that the majority of people consume fewer than the often recommended five portions of fruit and vegetables a day, since 78% (*n* = 90,448) consume fewer than five portions on a day where at least one portion is consumed, and not everyone consumes at least one portion every day (Fig. 2). The UK Department of Health introduced the ‘five-a-day’ campaign in 2003 following mounting evidence from health research, and a recommendation from the World Health Organization to consume a minimum of 400 g of fruit and vegetables per day to prevent chronic disease and micronutrient deficiencies (http://www.who.int/dietphysicalactivity/fruit/en/ last accessed on 3rd July 2018). Similar campaigns have been adopted in other major developed countries. Given the tendency for people to over-report perceived good behaviours in household surveys (see Lusk and Norwood, 2009a, Lusk and Norwood, 2009b), it seems likely that these figures represent an upper bound when it comes to the proportion of the population who regularly consume five portions of fruits and vegetables per day. This trend is broadly consistent with the findings from Australia by Mujcic and Oswald (2016), who report that 85% of respondents consumed fewer than 3 daily portions of fruit, and 60% consumed fewer than 3 daily portions of vegetables (though a direct comparison is not possible due to a difference in our measures of the quantity of fruit and vegetable consumption). 90 people consumed 11 or more portions of fruit and vegetables on a day where at least one portion was consumed. We recoded the data for the subsequent regression analysis, so that anyone consuming greater than 11 portions is assigned a value of 11. Recoding does not change the results of our analyses.

Fig. 2 shows the frequency of fruit and vegetable consumption in a usual week: 50% of people consume at least one portion of vegetables daily, and 46% of people consume at least one portion of fruit daily. Somewhat surprisingly, therefore, there is a fairly large proportion of people who go one day or more than a week consuming either no fruit, or no vegetables. 9310 individuals (7%) report never consuming fruit, and 2710 individuals (2%) report never consuming vegetables in a usual week.

#### 3.1.1 Income

**Fig. 3:**
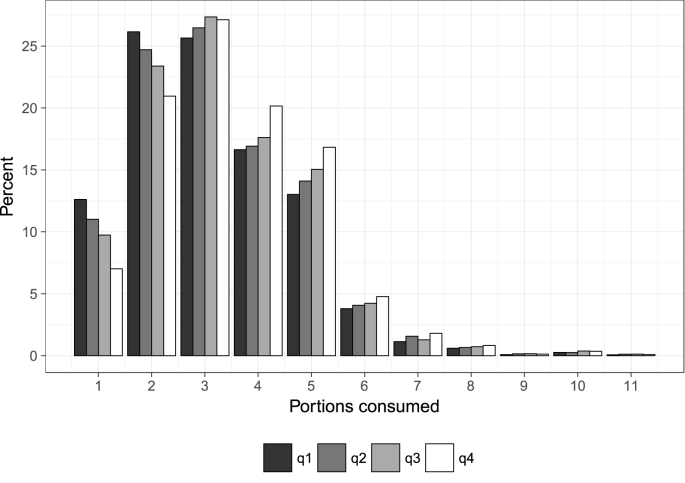
The number of portions of fruits and vegetables consumed on a day with positive consumption, separated by income quartile (notes: q1 represents the lowest income quartile; 11 portions means ’11 or more’; *n* = 90,361).

**Fig. 4:**
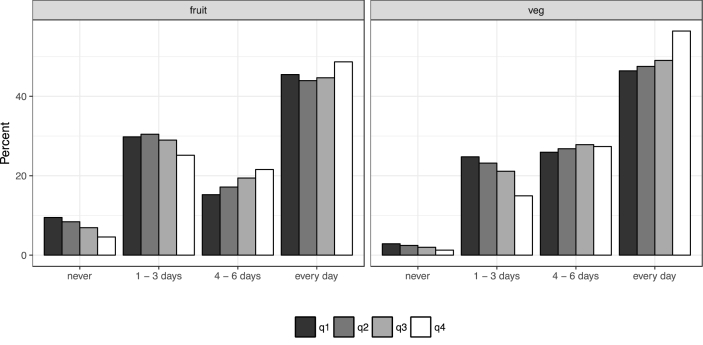
The frequency of fruit and vegetable consumption in a usual week, separated by income quartile (q1 represents the lowest income quartile; *n* = 130,437 for fruit, *n* = 130,447 for vegetables).

Here, we examine to what extent fruit and vegetable consumption differs according to three main socio-demographic characteristics. First, we separate individuals into income quartiles based on their household income in the month before the survey interview. Mean monthly household income in the sample is £3380 and median monthly household income is £2744. Fig. 3 shows graphs of portions consumed per day separated by income quartile. In general, the consumption distribution is quite similar across quartiles, albeit we do observe some income differences, that is, the higher the income quartile, the lower the proportion of individuals that consume less than the recommended five portions on a day where at least one portion is consumed (81%, 79%, 78%, and 75% for quartiles 1, 2, 3, and 4 respectively). A broadly similar pattern emerges for the frequency of consumption, in that we observe that those in the highest income quartile have a somewhat higher frequency of consumption than those in the lower income quartiles (Fig. 4). Again, the differences across income groups are not very substantive.

In short, as Fig. 3, Fig. 4 illustrate, those in the highest income quartile as a whole consume more fruits and vegetables than those in the lower income groups. These differences across the income distribution are, however, not very substantive; and in general terms, the percentage of individuals who do not consume five portions of fruits and vegetables (on a day where at least one portion is consumed) is high across the board. This suggests that being unable to afford fruits and vegetables in large quantities is perhaps not the main reason that levels of consumption are below recommended guidelines for the majority of the population. It is worth noting that a recent study has suggested that the scarcity of time can act independently to the scarcity of income in predicting poor eating habits (Venn and Strazdins, 2017), and therefore it could also be a factor in these data.

#### 3.1.2 Age

Next, we look at the differences in fruit and vegetable consumption across age. The mean age in our overall sample is 47.1 (*min* = *15, max* = *104*). Grouping the data into age bands based on 5 approximate quintiles, we find that approximately: 87% of 15–29 year-olds; 80% of 30–41 year-olds; 77% of 42–52 year-olds; 73% of 53–65 year-olds; and 74% those aged 66 and above consume fewer than the recommended five portions a day (on a day where there is non-zero consumption). In other words, up until approximately the age of retirement, an upward trend is evident when it comes to consumption of fruits and vegetables.

**Fig. 5:**
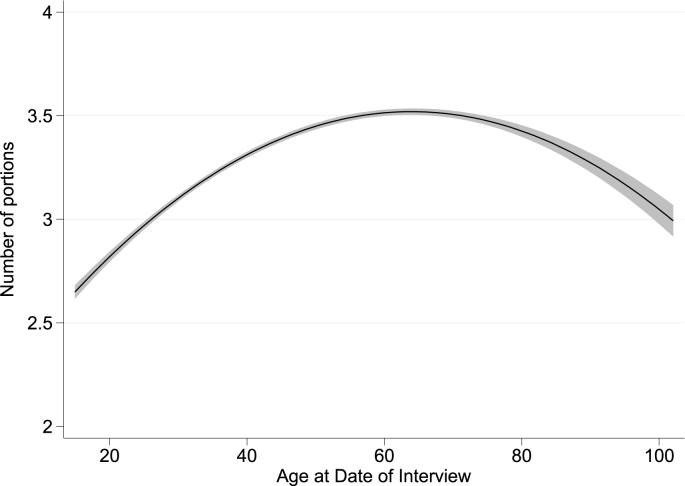
Fitted quadratic, with 95% confidence interval, showing the non-linear relationship between age and portions of fruits and vegetables consumed on a day where at least one portion is consumed (*n* = 90,445).

Closer inspection of the data in the oldest age group reveals an increasing proportion of people consuming fewer than five-a-day, on a day where at least one portion is consumed. For example, the proportion is 71% for 66-75 year-olds, but rises to 77% for 76-85 year-olds, and 84% for those aged 86 or above. This is in contrast to the falling proportion of individuals consuming fewer than five-a-day observed up until the age of 65. Suspecting a non-linear relationship, we plotted a fitted quadratic function to the raw data on the number of fruit and vegetable portions consumed (illustrated in Fig. 5). Looking at this figure, we can see that there appears to be a hump-shaped relationship between age and the quantity of fruits and vegetables consumed (on a day where at least one portion is consumed). The maximum consumption of an average 3.52 portions per positive consumption day is achieved at age 64, which is just before the normal state pension age in the UK (this age is currently variable between 65 and 68 depending on sex and birth year). While the quantity of fruit and vegetable consumption is rising slowly up to this point (i.e., people generally appear to be increasing the number of portions of fruits and vegetables they consume as they age), beyond the age of 64 we can see that the consumption of fruit and vegetables begins to fall. One potential explanation is that retirement leads to a reduced calorie intake because of lower activity levels and this reduced calorie intake, in turn, leads to a reduction in the quantity of fruits and vegetables consumed (on a day with positive consumption).

**Fig. 6:**
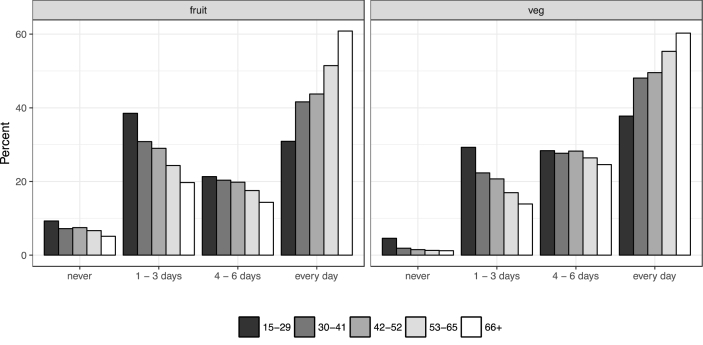
The frequency of fruit and vegetable consumption in a typical week, separated by age band (*n* = 130,540 for fruit; *n* = 130,550 for vegetables).

While the quantity of fruit and vegetables consumed starts to fall at retirement age, the frequency with which individuals consume fruit and/or vegetables continues to rise (Fig. 6). For example, the proportion of people eating at least one portion *every day* is increasing in age up until after the age of approximately 80. After the age of 80, whereas the proportion consuming one portion of *fruit* every day continues to increase, the proportion consuming one portion of *vegetables* every day levels off. One potential explanation for these seemingly contradictory findings is that individuals are simply more concerned about their health as they age (i.e., they consume more portions of fruit and vegetables and more frequently). However, upon retirement, the actual number of portions of fruit and vegetables consumed (on a day with a positive level of consumption) starts to decline as a result of not working (for example, due to a reduction in the overall number of calories expended). It is also possible that increasing health-related concerns surrounding the appropriate number of calories to consume could also play a role in reducing the number of portions consumed. We cannot, however, rule out other explanations such as cohort effects due to differences in upbringing and environment between generations.

#### 3.1.3 Sex

**Fig. 7:**
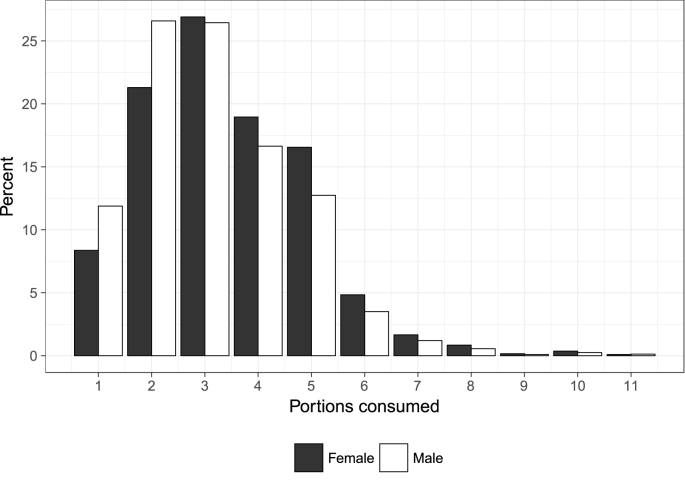
Sex differences in the quantity of fruit and vegetable portions consumed on a day where at least one portion is consumed (*n* = 90,448; 44.1% males).

**Fig. 8:**
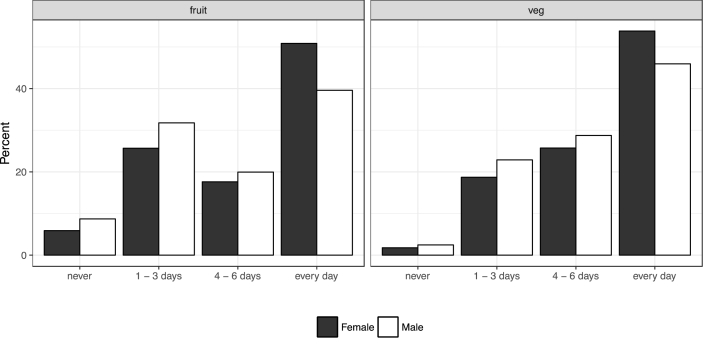
Sex differences in the frequency of fruit and vegetable consumption (*n* = 130,546 for fruit; *n* = 130,556 for vegetables).

Finally, we separate fruit and vegetable consumption by sex. Fig. 7 shows the differences in consumption (on days where at least one portion is consumed) between men and women. In keeping with previous research (e.g., see Lange et al., 2018), females eat significantly more portions of fruit and vegetables per positive consumption day (mean for females = 3.43; mean for males = 3.13; *p* < 0.00005). They also consume both fruits and vegetables more frequently throughout the week (Fig. 8).

There are two main ways to interpret these data. It is certainly plausible that women consume more fruits and vegetables than men in general. Previous research has found that women are more likely to be vegetarian, and more likely to be morally opposed to eating meat (Beardsworth et al., 2002; Ruby, 2012). Therefore, it is conceivable that female diets are more reliant on plant-based nutrition than male diets, which would support the suggestion that females have a higher level of fruit and vegetable consumption than males. However, we note that social desirability (the tendency to report differently based on what appears more desirable to others) is generally found to be higher in women. It has specifically been shown to bias dietary responses of women, but not men (Hebert et al., 1995, 1997). Hence, an alternative explanation is that women are simply over-reporting their consumption of fruit and vegetables.

#### 3.1.4 Socio-demographic regression

In Table A1, we present the results of a pooled cross-sectional model with fruit and vegetable consumption (on a day where at least one portion was consumed) as the dependent variable, and socio-demographic characteristics as our explanatory variables. The descriptive patterns described above hold in this analysis. Table A1 shows that the coefficient on age is positive (*p* < 0.001), whereas the coefficient for age-squared is negative (*p* < 0.001), indicating a hump-shaped relationship between age and the quantity of fruit and vegetables consumed. The coefficient for income attracts a positive and statistically significant coefficient (*p* < 0.001). Finally, we can see that females have a significantly higher level of consumption than males (*p* < 0.001). These relationships are robust to the addition of controls for education, employment, lifestyle and health, and the consumption of dairy and bread.

### 3.2 Regression analysis: GHQ-12

Our main results focus on the use of *reversed GHQ-12* as the measure of individuals' mental well-being. The key independent variable of interest is *fruit and vegetables* (the quantity of portions of fruits and/or vegetables on a typical day when consumption is non-zero). We first estimated a simple pooled OLS model, that is, a cross-sectional regression pooling data from multiple waves and treating them as distinct individuals. *Fruit and vegetables* attracts a positive and statistically significant coefficient in our pooled cross-sectional OLS model. In other words, as the quantity of fruit and vegetables consumed increases, then so too does well-being. Further details of these results appear in the appendix (Table A2).

Table 1 presents our main regression results for the full sample population in Britain, consisting of individual observations spread across two waves of the UKHLS. Here, instead of using a pooled cross-sectional model, we take advantage of the panel nature of the dataset by using individual fixed-effects, thereby controlling for any potential bias due to time-invariant unobserved heterogeneity (e.g., personality traits and family upbringing). In specification (1), a basic fixed effects regression of *mental well-being* on only *fruits and vegetables*, we see that *fruit and vegetables* attracts a statistically significant and positive coefficient. This suggests that when individuals increase their fruit and vegetable consumption, their mental well-being rises. In specification (2), we see that this coefficient remains relatively stable after controlling for standard socio-demographic variables. The coefficient in our fixed-effects specification is somewhat smaller than in our pooled cross-sectional model (Table A2), which suggests that, notwithstanding the standard set of socio-demographic controls, reliance on a cross-sectional model may place an upward bias on estimates of the relationship between fruit and vegetable consumption and well-being.

**Table 1:** Fixed-effects regression estimates showing the relationship between the quantity/frequency of fruit and vegetable consumption, and mental well-being, as measured using a reversed form of the GHQ-12 (*reversed GHQ-12*).

	Dependent variable: Reversed GHQ-12			
	(1)	(2)	(3)	(4)
Portions of fruit and veg per day (on a typical day when at least one portion is consumed)	0.116***	0.118***	0.133***	
	(0.0217)	(0.0220)	(0.0245)	
**Days each week eat fruit (reference is never)**				
1–3 days				0.259***
				(0.0896)
4–6 days				0.423***
				(0.0989)
Every day				0.613***
				(0.0982)
**Days each week eat vegetables (reference is never)**				
1–3 days				0.518***
				(0.171)
4–6 days				0.803***
				(0.175)
Every day				0.925***
				(0.177)
**Demographics**				
Age		−0.112	−0.115	−0.202**
		(0.136)	(0.148)	(0.0801)
Age^2^		0.00242***	0.00253***	0.00225***
		(0.000355)	(0.000405)	(0.000215)
Income (prev month)		0.00000258	0.00000477	0.0000109
		(1.39e-05)	(1.49e-05)	(9.53e-06)
Married		0.157	0.255	0.0266
		(0.184)	(0.193)	(0.119)
Divorced		−0.245	−0.231	−0.135
		(0.267)	(0.294)	(0.166)
Widowed		−0.591*	−0.682*	−0.825***
		(0.352)	(0.409)	(0.218)
Number of children		−0.0846	−0.103	−0.0325
		(0.0635)	(0.0686)	(0.0424)
**Employment status (reference is self-employed)**				
Employed		−0.310**	−0.367**	−0.185*
		(0.155)	(0.168)	(0.105)
Unemployed		−1.952***	−1.903***	−1.803***
		(0.203)	(0.220)	(0.139)
Inactive		−0.921***	−0.746***	−0.773***
		(0.172)	(0.185)	(0.117)
**Lifestyle/health**				
Currently a smoker			−0.247*	
			(0.145)	
Days walked ≥ 10 min			0.0175***	
			(0.00345)	
Long standing health condition			−0.568***	
			(0.0826)	
Dairy consumption dummies	No	No	Yes	No
Bread consumption dummies	No	No	Yes	No
Education dummies	No	Yes	Yes	Yes
Time (wave) dummies	No	Yes	Yes	Yes
Constant	24.44***	23.71***	23.77***	27.79***
	(0.0736)	(6.168)	(6.493)	(3.559)
Observations	79608	77913	66042	114560
*R*^*2*^	0.001	0.008	0.013	0.01
Number of unique individuals	52182	51456	45600	58137

Standard errors in parentheses. ***p < 0.01, **p < 0.05, *p < 0.1.

Notes: The four fixed-effects regressions in this table assess the impact of within-person changes in fruit and vegetable consumption on mental well-being, as measured by the *reversed GHQ-12*. (1) includes only a measure of the number of portions consumed on a day where at least one portion is consumed. (2) and (3) add variables for demographics, lifestyle/health, and consumption of bread/dairy. These three regressions use Waves 2 and 5 of the UKHLS. (4) uses the general form of (2), but measures fruit consumption frequency and vegetable consumption frequency across three Waves (2, 5, and 7). Additional controls on lifestyle/health and consumption of other foods were not available for all three Waves. All models show significant positive relationships between fruit and vegetable consumption, and mental well-being.

Specification (3) in Table 1 adds an additional set of covariates related to health and lifestyle behaviours. This includes type of dairy consumption, type of bread consumption, smoking status, walking frequency (the number of days over the last 4 weeks that individuals had completed a continuous walk lasting at least 10 min), and the presence of a long-standing health condition. These additional controls have the expected relationship with mental well-being. Specifically, we first observe a positive and significant association between walking frequency and well-being, which is in keeping with previous research on the benefits of physical activity on mental/psychological well-being (Scully et al., 1998; Fox, 1999; Penedo and Dahn, 2005). Second, we observe a negative and statistically significant relationship between smoking status and subjective well-being, which is also in keeping with previous work (e.g., Kahneman and Deaton, 2010; Shahab and West, 2012).

In relation to health status, we find as expected that individuals with a long-standing health condition report a significantly lower level of well-being than those without a long-standing health condition. As a robustness check, we also ran specification (3) with ‘long-standing health condition’ replaced by ‘general health,’ which is measured on a five-point scale ranging from *poor* to *excellent*. Our results relating to the relationship between fruit and vegetable consumption and well-being were robust to this alternative measure of health (results are available on request).

We observed no significant relationship between the type of bread or milk consumed and mental well-being. Apart from their use as proxy variables to capture other diet related health behaviours, their lack of statistical significance can be seen as adding further weight to our argument that fruit and vegetable consumption can foster mental well-being. That is, there appears to be something inherent in fruit and vegetables, as opposed to other food groups such as bread and milk, that positively influences individuals' well-being.

Looking at our preferred fixed-effects specification (3), we find that increasing one's consumption of fruit and vegetables by one portion (on a day where at least one portion is consumed) leads to a 0.133-unit increase in mental well-being (*p* < 0.01). The question remains: how large is this effect in practical terms? One way to gain an understanding of this issue is to compare the estimated change in mental well-being from increases in fruit and vegetable consumption to that of other commonly observed correlates with well-being. A five-portion increase in the number of fruits and vegetables consumed (on a day with positive consumption) would be associated with a 0.67-unit increase in mental well-being. From the coefficients in Table 1, we can see that this would be approximately equivalent in magnitude to the estimated well-being loss from widowhood (−0.68), and approximately one third of the estimated impact from unemployment, which is known to have one of the largest effects on subjective well-being (Clark and Oswald, 1994; Oswald, 1997; Di Tella et al., 2001; Frey and Stutzer, 2002; Blanchflower and Oswald, 2004; Dolan et al., 2008). In terms of a factor that is more controllable at the individual level, our results show that increasing one's daily consumption by one portion (on a day with positive consumption) provides the same estimated increase in mental well-being as 7.6 additional days of walking continuously for at least 10 min per 4 weeks.

While the preceding analysis examined the impact of the *quantity* of fruits and vegetables consumed on mental well-being, we are also able to examine the relationship between well-being and the *frequency* of fruit and vegetable consumption in a typical week. Specification (4) in Table 1 utilises the three waves of data that are available on frequency of fruit and vegetable consumption, and estimates the effects of both fruit consumption frequency and vegetable consumption frequency on mental well-being, including the socio-demographic controls from specification (2). As explained in the Method section, lifestyle and health variables were only present in two of the three waves of the UKHLS we use in this study. Therefore, in order to enable us to utilise the additional explanatory power of using three separate time points to examine variation within individuals, these variables were omitted in specification (4). Using specification (4), we find a monotonically increasing relationship: That is, the more often fruits and vegetables are consumed in a week, the higher mental well-being is likely to be. It is also notable that the effect of eating vegetables more often is somewhat larger than the estimated effect of eating fruit more often. The correlation between fruit consumption frequency and vegetable consumption frequency is 0.41. However, coefficient estimates remain relatively stable when including only a single measure of frequency (i.e., either fruit frequency or vegetable frequency) in the regression.

The estimated effects of increasing the frequency of fruit and/or vegetables consumed on mental well-being are comparable to that of many impactful life events. Two relative comparisons paint a telling picture. If an individual who consumed vegetables daily stopped consuming them altogether, they would suffer a greater estimated loss in mental well-being than becoming widowed, or approximately 57% of the loss of someone who went from being employed to being unemployed. One note of caution with this comparison is that we cannot say that this is a pure frequency effect – an individual may have both increased their frequency and quantity of consumption simultaneously.

### 3.3 Robustness check: life satisfaction

As a robustness check for our main results using mental well-being (*reversed GHQ-12)*, we repeated our analyses from Table 1 using self-reported life satisfaction as the dependent variable. To measure life satisfaction, respondents were shown the following statement: “*(Please choose the number which you feel best describes how dissatisfied or satisfied you are with the following aspects of your current situation.) Your life overall*.” Responses were scored on a 7-point scale, where one means ‘completely dissatisfied’ and seven means ‘completely satisfied’. The results from this secondary set of analyses are in Table 2. Overall, the results paint a similar picture to those obtained when using *reversed GHQ-12* as the outcome variable for well-being. That is, the quantity and frequency of fruit and vegetable consumption both have a significant and positive relationship with life satisfaction.

**Table 2:** Additional fixed-effects regression estimates for robustness, showing the relationship between the quantity/frequency of fruit and vegetable consumption, and life satisfaction.

	Dependent variable: Life satisfaction (1–7 scale)			
	(1)	(2)	(3)	(4)
Portions of fruit and veg per day (on a typical day when at least one portion is consumed)	0.0172***	0.0263***	0.0191***	
	(0.00646)	(0.00656)	(0.00736)	
**Days each week eat fruit (reference is never)**				
1–3 days				0.0323
				(0.0264)
4–6 days				0.0924***
				(0.0291)
Every day				0.117***
				(0.0289)
**Days each week eat vegetables (reference is never)**				
1–3 days				0.112**
				(0.0502)
4–6 days				0.136***
				(0.0515)
Every day				0.189***
				(0.0519)
**Demographics**				
Age		0.101**	0.0948**	0.0255
		(0.0405)	(0.0445)	(0.0236)
Age^2^		−0.0000495	0.0000536	0.000250***
		(0.000106)	(0.000122)	(6.32e-05)
Income (prev month)		1.22e-05***	1.06e-05**	6.49e-06**
		(4.14e-06)	(4.50e-06)	(2.80e-06)
Married		0.152***	0.104*	0.134***
		(0.0548)	(0.0583)	(0.0351)
Divorced		−0.0856	−0.169*	0.0176
		(0.0797)	(0.0885)	(0.0487)
Widowed		−0.166	−0.151	−0.133**
		(0.104)	(0.122)	(0.0639)
Number of children		0.00655	0.0206	−0.00539
		(0.0189)	(0.0206)	(0.0125)
**Employment status (reference is self-employed)**				
Employed		−0.0433	−0.0164	−0.0458
		(0.0462)	(0.0505)	(0.0310)
Unemployed		−0.296***	−0.217***	−0.310***
		(0.0605)	(0.0661)	(0.0408)
Inactive		−0.0572	0.0159	−0.0592*
		(0.0512)	(0.0558)	(0.0343)
**Lifestyle/health**				
Currently a smoker			−0.0189	
			(0.0435)	
Days walked ≥ 10 min			0.00351***	
			(0.00104)	
Long standing health condition			−0.101***	
			(0.0248)	
Dairy consumption dummies		No	Yes	No
Bread consumption dummies		No	Yes	No
Education dummies		Yes	Yes	Yes
Time (wave) dummies		Yes	Yes	Yes
Constant	5.092***	0.647	1.035	3.252***
	(0.0219)	(1.838)	(1.956)	(1.047)
Observations	79610	77910	66035	114691
*R*^*2*^	0.0086	0.018	0.019	0.014
Number of unique individuals	52155	51425	45566	58149

Standard errors in parentheses. ***p < 0.01, **p < 0.05, *p < 0.1.

Notes: The four fixed-effects regressions in this table assess the impact of within-person changes in fruit and vegetable consumption on subjective well-being, as measured by *life satisfaction*. (1) includes only a measure of the number of portions consumed on a day where at least one portion is consumed. (2) and (3) add variables for demographics, lifestyle/health, and consumption of bread/dairy. These three regressions use Waves 2 and 5 of the UKHLS. (4) uses the general form of (2), but measures fruit consumption frequency and vegetable consumption frequency across three Waves (2, 5, and 7). The results in this table provide robustness to those in Table 1, which uses *reversed GHQ-12* as the dependent variable to find similar positive relationships.

In our preferred fixed-effects specification (3), the presence of a long-standing health condition generates a loss in life satisfaction equivalent to consuming 5.3 fewer portions of fruit and vegetables a day (on a day when there is non-zero consumption). Increasing consumption by one portion on a day when at least one portion is consumed increases life satisfaction by as much as walking continuously for at least 10 min on 5.4 additional days per 4 weeks. Finally, increasing the frequency of vegetable consumption from never to 4–6 days per week generates approximately the same estimated increase in life satisfaction as being married, whereas moving in the opposite direction (reducing consumption from 4 to 6 days per week to never) generates approximately the same estimated loss in life satisfaction as being widowed.

## 4 Discussion

This study contributes to the relatively new line of literature assessing the impact of fruit and vegetable consumption on well-being by being the first to analyse this relationship using the UK Understanding Society longitudinal dataset. One additional novel feature of our work is that we illustrate how the *frequency* with which fruits and vegetables are consumed are as important, if not more so, than actual *quantity* of consumption. To the best of our knowledge, this study is also the first to show a significant non-linear relationship between age and fruit and vegetable consumption.

Using fixed-effects analysis on over 45,000 individuals followed over time, we find that well-being increases in a dose-response fashion with the number of portions of fruit and vegetables consumed (on a day where there is non-zero consumption of fruit and vegetables) or with the number of days in which either fruits or vegetables are consumed in a given week. This finding is robust to the measure of well-being used (*reversed GHQ-12* and life satisfaction).

The importance of better understanding ways to improve mental health is an issue that has gained increasing prominence among public health professionals in the Western world. Specifically, there is a realisation of the importance of not just physical but also mental and subjective well-being. Apart from being significant in a statistical sense, our estimates suggest that even modest increases in the consumption of fruit and vegetables may have substantive positive effects on well-being. Therefore, when it comes to improving mental health, policies aimed at increasing fruit and vegetable consumption among the general population may provide a relatively low-cost public health intervention that supplements current approaches (generally involving pharmaceuticals and/or cognitive behavioural therapy).

It is worth noting at this stage that our life satisfaction estimates are broadly similar to those reported by Mujcic and Oswald (2016) using data from Australia. Given that our measure of fruit and vegetable consumption pertains to levels of consumption on a day when individuals eat at least one portion, as opposed to average daily levels per say, our estimates cannot be directly compared. However, there is a degree of similarity between our estimates of the well-being gains (as captured by life satisfaction) from increasing fruit and vegetable consumption, and those reported by Mujcic and Oswald (2016). Their estimates suggest, for example, that an increase in fruit and vegetable portions by eight per day would lead to the same average estimated life satisfaction gain as moving from unemployment to employment. Our findings suggest that moving from unemployment to employment has an equivalent effect on life satisfaction as increasing consumption of fruits and vegetables by approximately 10.5 portions per day on a typical day where some fruits and vegetables are consumed.

### 4.1 Limitations and considerations for future research

Future research is needed to help deal with the vexed issue of causality. That being said, it is important to note that our use of panel data coupled with a large representative survey and a detailed set of controls does, at least in part, address endogeneity concerns. Specifically, our approach helps ensure that any observed relationship between fruit and vegetable consumption and well-being is not merely a spurious cross-sectional pattern caused by omitted confounding variables such as personality traits, health related behaviours, or family upbringing. While it seems more likely that fruit and vegetable consumption increases subjective well-being, we recognise that the reverse may also be possible, at least to some extent, and it would place an upward bias on our estimates. Conversely, relying on self-reported measures of fruit and vegetable intake will inevitably introduce some measurement error, a factor that would typically bias our estimates downwards. We therefore suggest that additional research using longitudinal data (as used in our study) is still needed, but is also supplemented with randomised control trials (RCTs) and/or quasi-experimental methods (e.g., instrumental variable modelling) designed to explore the impact of different kinds of diets on well-being. An instrumental variable strategy would require identification of a variable strongly related to fruit and vegetable consumption, but not well-being. RCTs will likely require government involvement, but the rewards from such research promise great potential savings in terms of public expenditure on improving mental well-being.

Further research on the mechanisms in which these effects take place is also warranted. The epidemiological literature points to a number of potential mechanisms supportive of a positive relationship between fruit and vegetable consumption and subjective well-being, but the potential pathways are still poorly understood. Some possible mechanisms here include the role of vitamins for subjective well-being (see Kaplan et al., 2007 for a review). For example, the antioxidant properties in vitamin C and E have been shown to help manage the body's level of oxidative stress and lower inflammatory markers which have been associated with the onset of depressive mood (Berk et al., 2013; Rooney et al., 2013). B-vitamins play an essential role in maintaining mitochondrial function and mitochondrial dysfunction is associated with stress, anxiety, and social subordination (Chakravarty et al., 2013; Depeint et al., 2006; Einat et al., 2005; Hollis et al., 2015). Fruits and vegetables tend to be carbohydrate dense and there is some research to suggest that the positive effects of fruit and vegetable consumption could also be partly due to increased carbohydrate intake, as carbohydrate-rich foods increase concentrations of brain serotonin (Blundell et al., 1995; Takeda et al., 2004). It is also possible that not all carbohydrates affect well-being in the same fashion (White et al., 2013). Complex carbohydrates found in fruits and vegetables may enhance positive affect, whereas refined sucrose more commonly found in sweets and sugary soft drinks may worsen mood (Christensen and Burrows, 1990; Reid et al., 2010).

An alternative mechanism relies on substitution effects. Given that there is an upper bound to the number of calories an individual can comfortably consume per day, consuming more fruits and vegetables may result in reduced consumption of other food groups that may be detrimental to well-being. We suggest that future survey designs include measures for the consumption of other foods (e.g., meat, sweets, sugary drinks), as well as overall calories consumed. This would enable us to further disentangle the intrinsic well-being benefits of fruits and vegetables from substitution effects.

## 5 Conclusions

In conclusion, our study using panel data from the UK suggests that mental well-being responds in a dose-response fashion to increases in both the quantity and the frequency of fruits and vegetables consumed. These findings in turn could have important implications for public health practitioners, especially given the low rate of adherence to the national ‘five-a-day’ guidelines, as it suggests that even modest changes in the consumption patterns of individuals may translate into substantive positive effects for the well-being of large cohorts of the population. The relatively low rate of adherence to recommended guidelines points to the importance of further behavioural orientated research investigating how individuals make decisions about their diet, in order to help us understand how best to encourage higher levels of fruit and vegetable consumption. It is of course possible (if not likely) that by simply communicating the potential mental well-being benefits in addition to physical health benefits, one may be able to increase fruit and vegetable consumption. This strategy may be particularly effective due to the fact that positive effects for mental well-being accrue relatively rapidly, whereas the benefits in terms of physical health accrue in the medium to long-term.
