# Supplementary material for: Lettuce be happy: A longitudinal UK study on the relationship between fruit and vegetable consumption and well-being
Source: Soc Sci Med. 2019 Feb;222:335–45. doi: 10.1016/j.socscimed.2018.12.017 (PMC6381324; doi:10.1016/j.socscimed.2018.12.017)
Supplement: Online Data [file mmc1.pdf]

Online appendix for 'Lettuce be happy: A longitudinal UK study on the relationship between fruit and vegetable consumption and well-being'

Table A1: Additional pooled OLS regression estimates showing the cross-sectional relationship between fruit & vegetable consumption and demographic characteristics

Dependent variable: number of fruit and vegetable portions consumed (on a day where at least one portion consumed)		
	(1)	(2)
Age	0.0331***	0.0164***
	(0.00165)	(0.00201)
Age^2	-0.000223***	-4.12e-05**
	(1.61e-05)	(2.09e-05)
Male	-0.341***	-0.278***
	(0.0102)	(0.0111)
Income (prev month)	5.09e-05***	1.83e-05***
	(1.88e-06)	(2.06e-06)
**Other controls**		
Married	0.145***	0.115***
	(0.0161)	(0.0171)
Divorced	-0.0229	-0.00947
	(0.0233)	(0.0249)
Widowed	-0.143***	-0.143***
	(0.0277)	(0.0313)
Number of children	-0.0573***	-0.00163
	(0.00576)	(0.00616)
Education dummies	No	Yes
Employment dummies	No	Yes
Dairy and bread consumption dummies	No	Yes
Lifestyle and health dummies	No	Yes
Wave dummy	0.128***	0.0757***
	(0.0101)	(0.0107)
Constant	2.167***	1.703***
	(0.0354)	(0.0531)
Observations	90360	74177
R^2	0.049	0.134

Standard errors in parentheses. *** p<0.01, ** p<0.05, * p<0.1

Notes: These are pooled OLS regressions designed to test the relationship between demographic variables such as age, income, and sex, and the quantity of fruit and vegetable portions consumed on a typical day where at least one portion is consumed. Age appears to have a hump-shaped relationship with fruit and vegetable consumption, even after controlling for income. (1) only includes a short set of socio-demographic variables. (2) adds additional variables for education, employment, lifestyle, dairy consumption, and bread consumption, as in specification 3 of our main table.

Table A2: Pooled OLS regressions showing the cross-sectional relationship between daily fruit and vegetable consumption, and well-being

	Dependent Variable:			
	Reversed GHQ-12	*Reversed GHQ-12*	Life satis	Life satis
Portions of fruit and veg per day (on a typical day when at least one portion is consumed)	0.255***	0.252***	0.0897***	0.0747***
	(0.0127)	(0.0130)	(0.00342)	(0.00350)
**Demographics**				
Age		-0.205***		-0.0704***
		(0.00699)		(0.00188)
Age^2		0.00229***		0.000759***
		(7.12e-05)		(1.92e-05)
Male		0.979***		-0.0044
		(0.0405)		(0.0109)
Income (prev month)		7.24e-05***		3.35e-05***
		(7.59e-06)		(2.04e-06)
Married		0.196***		0.264***
		(0.0632)		(0.0170)
Divorced		-0.928***		-0.188***
		(0.0907)		(0.0244)
Widowed		-0.452***		-0.0252
		(0.110)		(0.0296)
Number of children		0.0408*		-0.00616
		(0.0228)		(0.00615)
**Education (reference category is no qualification)**				
Degree		0.685***		0.228***
		(0.0751)		(0.0202)
Other higher degree		0.626***		0.169***
		(0.0816)		(0.0220)
A-Level etc		0.474***		0.115***
		(0.0742)		(0.0200)
GCSE etc		0.565***		0.0981***
		(0.0726)		(0.0196)
Other qualification		0.331***		0.00548
		(0.0830)		(0.0224)
**Employment Status (reference category is self-employed)**				
Employed		-0.113		0.0197
		(0.0767)		(0.0206)
Unemployed		-2.580***		-0.597***
		(0.115)		(0.0310)
Inactive		-1.605***		-0.142***
		(0.0843)		(0.0227)
Wave dummy		-0.103***		-0.193***
		(0.0391)		(0.0105)
Constant	23.98***	27.42***	4.851***	6.054***
	(0.0466)	(0.186)	(0.0126)	(0.0500)
Observations	79608	77913	79610	77910
R^2	0.005	0.05	0.009	0.058

Standard errors in parentheses. *** p<0.01, ** p<0.05, * p<0.1

Notes: The four regressions in this table are pooled OLS regressions that measure the cross-sectional relationship between the number of fruit and vegetable portions consumed (on a day where at least one portion is consumed), and well-being. The first two regressions use *reversed GHQ-12* as a measure of mental well-being. The third and fourth regressions use *life satisfaction* (measured on a 1-7 scale) as a measure of subjective well-being. All regressions show a positive relationship.
